# Parkinson’s Spectrum Mechanisms in Pregnancy: Exploring Hypothetical Scenarios for MSA in the Era of ART

**DOI:** 10.3390/ijms26073348

**Published:** 2025-04-03

**Authors:** Dalibor Kovacevic, Gordana Velikic, Dusan M. Maric, Dusica L. Maric, Miljan Puletic, Ljiljana Gvozdenovic, Danilo Vojvodic, Gordana Supic

**Affiliations:** 1 “Genesis” Hospital, 21000 Novi Sad, Serbia; 2 Department for Research and Development, Clinic Orto MD-Parks Dr Dragi Hospital, 21000 Novi Sad, Serbia; 3 Hajim School of Engineering, University of Rochester, Rochester, NY 14627, USA; 4 Faculty of Stomatology Pancevo, University Business Academy, 26000 Pancevo, Serbia; 5 Department of Anatomy, Faculty of Medicine, University of Novi Sad, 21000 Novi Sad, Serbia; 6 Department of Anesthesia, and Intensive Care, Clinical Center Vojvodina, Faculty of Medicine, University of Novi Sad, 21000 Novi Sad, Serbia; 7 Institute for Medical Research, Military Medical Academy, 11000 Belgrade, Serbia; 8 Medical Faculty of Military Medical Academy, University of Defense, 11000 Belgrade, Serbia

**Keywords:** multiple system atrophy, pregnancy, hormonal changes, Parkinson’s spectrum, assisted reproductive technologies, α-synuclein, microchimerism, epigenetics

## Abstract

Multiple System Atrophy (MSA) is a rare, rapidly progressive neurodegenerative disorder marked by autonomic dysfunction, parkinsonism, and cerebellar ataxia. While predominantly affecting individuals in their fifth or sixth decade, advancements in assisted reproductive technologies (ART) have created new clinical scenarios involving pregnancies in women within MSA’s typical onset range. Given the scarcity of documented MSA pregnancies, this review leverages insights from related Parkinson’s spectrum mechanisms to explore hypothetical scenarios for how pregnancy-induced physiological changes might influence MSA progression. Pregnancy-induced hormonal fluctuations, including elevated estrogen and progesterone levels, may modulate α-synuclein aggregation and neuroinflammatory pathways. Immune adaptations, such as fetal microchimerism and Th2-biased immune profiles, introduce additional complexities, particularly in donor embryo pregnancies involving complex microchimerism. Metabolic demands and oxidative stress further intersect with these mechanisms, potentially accelerating disease progression. We analyze existing literature and theoretical models, emphasizing the need for interdisciplinary research. Clinical implications are discussed to propose evidence-based strategies for optimizing maternal-fetal outcomes. This paper identifies critical knowledge gaps and proposes avenues for future investigation to optimize maternal-fetal outcomes in this unique and underexplored clinical intersection.

## 1. Introduction

Multiple System Atrophy (MSA) is a rare, fatal, neurodegenerative disorder with limited diagnostic and therapeutic options, and 50–60 years as typical range of onset. The disease is marked by the accumulation of misfolded α-synuclein (α-syn) protein within oligodendrocytes, leading to widespread neural degeneration and a decline in motor and autonomic functions. Diagnosing MSA is particularly challenging due to its symptomatic overlap with other neurodegenerative conditions such as Parkinson’s disease (PD) and pure autonomic failure. Given the overlap in some pathophysiological mechanisms, MSA is considered part of the broader Parkinson’s spectrum. The absence of definitive biomarkers and the lack of disease-modifying therapies further complicate clinical management, making MSA one of the most formidable disorders in neurology. However, due to limitations in sensitivity and specificity of current biomarker-based diagnostics, MSA may remain undetected or be misdiagnosed at ages younger than the typical onset range. This diagnostic uncertainty further underscores the urgent need for increased research and clinical attention to this disorder.

Pregnancy induces profound physiological changes, including hormonal fluctuations, immune modulation, and metabolic adaptations, to support fetal development and prepare the maternal body for childbirth. While neurodegenerative diseases predominantly affect individuals beyond reproductive age, advancements in assisted reproductive technologies (ART) have shifted the maternal age range upward, allowing pregnancies to occur within the typical onset range of MSA. Although the intersection between ART-enabled pregnancies and early-onset or prodromal MSA is currently rare, increasing sensitivity and diagnostic accuracy could result in more frequent recognition of such cases, making exploration of this scenario clinically relevant. This shift and increasing accuracy in diagnostic tools raise questions about how pregnancy-induced physiological changes, such as hormonal fluctuations, immune adaptations, and metabolic stress, might interact with MSA pathophysiology. To address this, we extrapolate insights from Parkinson’s spectrum mechanisms, particularly those documented in PD and related synucleinopathies, to explore plausible scenarios for MSA during pregnancy. These mechanisms provide a foundation for understanding how hormonal and immune adaptations in pregnancy might influence α-syn aggregation, neuroinflammation, and neurodegeneration.

This review aims to fill critical knowledge gaps by synthesizing existing evidence and proposing hypotheses for MSA progression during pregnancy. By integrating insights from related fields and identifying research priorities, this work lays the groundwork for future studies and clinical strategies to optimize outcomes for this unique patient population.

### Glossary and Comparative Context

Considering the multidisciplinary angle of this topic and aiming to enhance accessibility for a broader audience, we have included a glossary—see Table 1—defining key terms essential for understanding the complex interactions discussed in this paper.

Given the rarity of documented cases of MSA during pregnancy, understanding potential impacts largely relies on insights drawn from related neurodegenerative conditions with better-documented interactions with pregnancy, such as PD [1] and multiple sclerosis (MS) [2]. For instance, MS is well-known for its pregnancy-related modulation, typically presenting reduced relapse rates during gestation, providing a model of immune modulation relevant to neurological disorders. Similarly, the PD literature offers evidence of hormonal modulation effects on dopaminergic systems, potentially applicable to understanding how similar mechanisms might operate in MSA. Throughout this manuscript, we utilize comparative references from Parkinson’s spectrum research to strengthen the hypotheses discussed and suggest plausible biological interactions in MSA cases occurring during pregnancy.

## 2. MSA Pathophysiology

### 2.1. Molecular Biology Aspects

**Figure 1:**
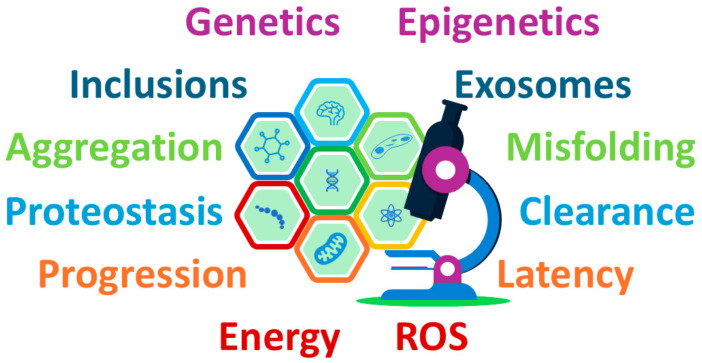
Molecular and cellular mechanisms in MSA pathophysiology—the fundamental mechanisms implicated in the pathophysiology of MSA include α-syn aggregation and toxic species formation, the development of glial cytoplasmic inclusions in oligodendrocytes, and disruptions in cellular energy pathways driven by mitochondrial dysfunction. Additionally, genetic and epigenetic factors, protein aggregation and misfolding pathways (proteostasis and clearance), along with the role of ROS (reactive oxygen species) and exosomes, and the interplay between neurodegeneration progression and latency are highlighted as interconnected contributors to disease development.

Figure 1 highlights the key mechanisms implicated in MSA pathophysiology, including α-syn aggregation, glial cytoplasmic inclusions, and mitochondrial dysfunction. These interconnected processes form the foundation for understanding the progression of neurodegeneration in MSA and are explored in detail below.

#### 2.1.1. α-Syn Aggregation and Glial Cytoplasmic Inclusions

MSA is neuropathologically characterized by the accumulation of misfolded α-syn proteins within oligodendrocytes, forming glial cytoplasmic inclusions (GCIs). α-syn is a 140-amino acid presynaptic neuronal protein implicated in synaptic vesicle regulation and neurotransmitter release [3,4]. In MSA, α-syn undergoes abnormal aggregation due to misfolding, post-translational modifications, e.g., phosphorylation at serine-129, and impaired degradation pathways. The formation of GCIs is intrinsic to MSA and distinguishes it from other synucleinopathies like PD, where predominant ones are neuronal inclusions called Lewy bodies. The exact mechanism leading to α-syn accumulation in oligodendrocytes remains unclear. One hypothesis suggests that oligodendrocytes might aberrantly express α-syn, or alternatively, they may internalize extracellular α-syn released from neurons through exosomes or endocytosis. The aggregated α-syn in GCIs disrupts oligodendroglial function, leading to demyelination and subsequent neuronal degeneration. Demyelination impairs the conduction velocity of action potentials, resulting in motor and autonomic dysfunctions characteristic of MSA. Additionally, GCIs can induce endoplasmic reticulum (ER) stress, mitochondrial dysfunction, and activation of apoptotic pathways in oligodendrocytes [5,6].

**Figure 2:**
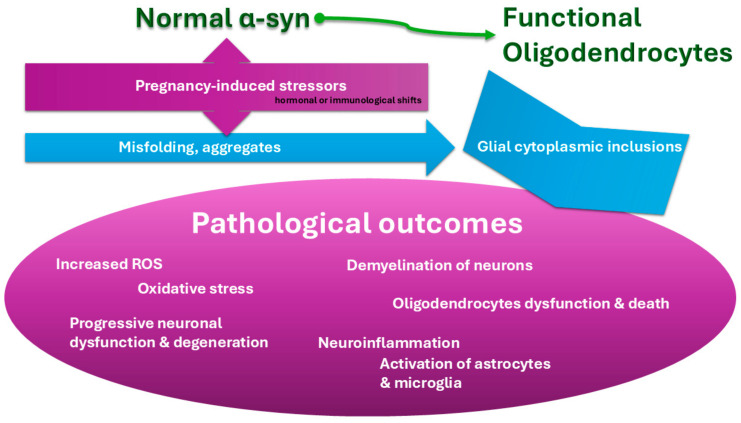
α-syn aggregation and pathological outcomes. Flow diagram representing how α-syn accumulation affects oligodendrocytes, leading to MSA pathology, potentially accelerated by pregnancy-induced stress factors.

MSA shares overlapping mechanisms with other synucleinopathies, such as PD, including α-syn aggregation and neuroinflammation. While these mechanisms are less understood in MSA, findings from Parkinson’s spectrum research provide a framework for hypothesizing how pregnancy-related physiological changes might interact with MSA pathophysiology. See Figure 2 for simplified presentation of α-syn aggregation and neuroinflammation process.

#### 2.1.2. Neurodegeneration Mechanisms and Genetic and Epigenetic Factors Relevant to MSA

The neurodegeneration observed in MSA involves a multifactoral connection of cellular and molecular processes, with limited genetic implication. Table 2 displays neurodegeneration mechanisms, known genetic predispositions, and epigenetic modifications relevant to MSA [7,8,9].

Aggregated α-syn impairs mitochondrial function by disrupting electron transport chain complexes, leading to reduced ATP production and increased reactive oxygen species (ROS) generation [5]. Mitochondrial DNA mutations and defects in mitochondrial dynamics further exacerbate cellular energy deficits. Elevated ROS levels cause oxidative damage to lipids, proteins, and nucleic acids. Oxidative stress activates signaling pathways that promote neuroinflammation and apoptosis. Markers of lipid peroxidation and protein carbonylation are elevated in MSA brain tissues. Activated microglia and astrocytes release pro-inflammatory cytokines, e.g., TNF-α, IL-1β and chemokines, contributing to a chronic inflammatory milieu. Noticeably, the NF-κB pathway is upregulated in MSA, driving the expression of these cytokines and creating a feed-forward loop of neuroinflammation. Modulating NF-κB activity through pharmacological or genetic approaches could offer experimental targets for reducing inflammatory damage in MSA [11,12,13].

Neuroinflammation can exacerbate neuronal injury and promote further α-syn aggregation through the release of ROS and nitric oxide. α-syn aggregation can be compared to a “traffic jam within brain cells”, obstructing normal cellular processes, leading to neurological impairment. Dysfunctional ubiquitin-proteasome and autophagy-lysosome pathways hinder the clearance of misfolded proteins. The accumulation of dysfunctional proteins overwhelms cellular quality control mechanisms, leading to proteotoxic stress. Abnormal iron accumulation has been observed in the basal ganglia of MSA patients [10]. Iron can catalyze the formation of hydroxyl radicals via the Fenton reaction, amplifying oxidative stress and neuronal damage. Misfolded α-syn may propagate in a prion-like manner, spreading from affected to healthy cells, via mechanisms such as exosome release, tunneling nanotubes, or direct membrane contact. Exosomes released in neuroinflammatory states are enriched with pathogenic α-syn, and targeting exosomal pathways could provide a novel therapeutic avenue to halt disease progression [14].

MSA is predominantly sporadic, with few familial cases reported, suggesting a limited genetic contribution. However, several genetic factors have been investigated. The *SNCA* gene encodes α-syn [9,15], and single nucleotide polymorphisms (SNPs) in the *SNCA* locus have been associated with increased risk for MSA in genome-wide association studies (GWAS). These variants may influence α-syn expression levels or aggregation propensity. Mutations in the *COQ2* gene, involved in coenzyme Q10 biosynthesis, have been linked to MSA in Japanese cohorts. *COQ2* mutations may lead to mitochondrial dysfunction due to impaired electron transport. Copy number variations (CNVs) affecting genes involved in proteostasis and autophagy may contribute to disease susceptibility by altering gene expression. Variants in the microtubule-associated protein tau (MAPT) gene and other genes related to mitochondrial function, oxidative stress, and neuroinflammation have been explored, but findings are inconsistent across populations.

Epigenetic mechanisms regulate gene expression without altering the DNA sequence and may play a role in MSA pathogenesis. Aberrant methylation patterns can silence or activate genes implicated in neurodegeneration. Hypomethylation of the *SNCA* promoter region may upregulate α-syn expression. Genome-wide methylation studies have identified differentially methylated regions in MSA patients compared to controls. Post-translational modifications of histones, such as acetylation, methylation, and phosphorylation, influence chromatin structure and gene accessibility. Histone deacetylase (HDAC) inhibitors have shown neuroprotective effects in preclinical models by enhancing the expression of neuroprotective genes and reducing neuroinflammation. In particular, HDAC6 inhibition has demonstrated promise in restoring mitochondrial function and reducing α-syn aggregation in cellular models, suggesting this could be a viable therapeutic pathway for MSA.

MicroRNAs (miRNAs) and long non-coding RNAs (lncRNAs) can modulate gene expression post-transcriptionally. Dysregulated miRNAs have been implicated in α-syn aggregation, mitochondrial dysfunction, and inflammatory responses. For example, miR-155 has been shown to regulate pro-inflammatory pathways, while its inhibition reduces microglial activation and ROS production, providing a potential target for modulating neuroinflammation in MSA [16,17,18]. Exposure to environmental toxins such as pesticides and heavy metals and lifestyle factors such as diet and stress, can induce epigenetic changes that may predispose individuals to MSA. Epigenetic modifications may be a link between environmental factors and genetic susceptibility [10,14,15]. Exploring interactions between these epigenetic changes and established MSA risk genes, such as *SNCA* and *COQ2*, could provide deeper insight into the disease’s multifactorial nature.

## 3. Changes During Pregnancy

### 3.1. Endocrine Adaptations

Pregnancy induces profound hormonal changes to support fetal development and prepare the maternal body for childbirth, which involves significant hormonal fluctuations, including increases in estrogen and progesterone [19]. These hormones can influence neurological conditions through their effects on the autonomic nervous system, which is heavily impacted in MSA. Estrogen promotes uterine growth by enhancing uteroplacental blood flow, and modulating lipid and protein metabolism. Primarily produced by the placenta, estrogen levels increase dramatically, reaching up to 100-fold levels. Progesterone maintains the endometrial lining while inhibiting uterine contractions and modulating the immune response to prevent fetal rejection [20]. These hormonal changes also modulate epigenetic pathways, including DNA methylation and histone modifications, which may alter the expression of genes implicated in MSA pathogenesis, such as *SNCA*. For instance, the increased levels of estrogen during pregnancy could either enhance neuroprotection or exacerbate α-syn aggregation through differential epigenetic regulation.

Human Chorionic Gonadotropin (hCG) produced by the trophoblasts, peaks during the first trimester and supports the corpus luteum to sustain progesterone production until the placenta takes over. Human Placental Lactogen (hPL) influences maternal glucose metabolism, promoting lipolysis and increasing free fatty acids to meet fetal energy demands. Oxytocin levels increase towards the end of pregnancy, facilitating uterine contractions during labor and promoting milk ejection during lactation. Prolactin levels rise to prepare the mammary glands for milk production. Relaxin is secreted to relax pelvic ligaments and soften the cervix in preparation for childbirth [21,22].

### 3.2. Impact of Hormonal Changes on the Nervous System

The elevated hormones during pregnancy have significant effects on the maternal nervous system. Estrogen and progesterone influence microRNA expression, including miR-155, which is involved in inflammatory pathways. During pregnancy, dysregulated miRNA profiles could amplify neuroinflammatory responses, contributing to disease progression in women with MSA. Targeting these miRNAs may represent an avenue for mitigating pregnancy-related exacerbation of MSA symptoms.

Hormonal influences on the nervous system operate through multiple interconnected mechanisms, with estrogen serving as a prime example of neuroactive steroid action. Its neuroprotective properties manifest through several key pathways that maintain neural health and function. The reduction in oxidative stress occurs through direct antioxidant actions and enhancement of mitochondrial function, particularly in regions with high metabolic demands such as the hippocampus and brainstem autonomic centers. This hormone optimizes mitochondrial energy production while preventing excessive free radical formation, thereby supporting neuronal resilience [22].

In terms of neuronal survival pathways, estrogen activates rapid non-genomic signaling through membrane-associated estrogen receptors, such as mERα and mERβ. It stimulates the PI3K/Akt and MAPK pathways, crucial for neuronal survival, and increases expression of antiapoptotic proteins while suppressing proapoptotic factors [23,24].

Estrogen modulates synaptic plasticity by influencing dendritic spine morphology and increasing its density specifically in areas crucial for cognitive function and auto-nomic regulation, such as in hippocampal neurons and prefrontal cortex, while upregu-lating NMDA receptor subunit expression, particularly NR2B [25]. It regulates the expression and trafficking of neurotransmitter receptors while promoting the synthesis of synaptic proteins essential for neural communication and adaptation, including PSD-95 and synaptophysin, and modulates AMPA receptor trafficking and function [26,27,28].

The interaction with major neurotransmitter systems demonstrates estrogen’s broad influence on neural function. It modulates dopaminergic transmission, affecting motor control and reward circuitry. Serotonergic system modification influences mood regulation and autonomic function, while cholinergic system modulation impacts attention and memory processes. These effects extend beyond simple neurotransmitter level adjustments to include changes in receptor density, transporter function, and synaptic release mechanisms [27,28].

The hormone’s immunomodulatory role in the nervous system involves sophisticated regulation of inflammatory processes. Through the downregulation of pro-inflammatory cytokines such as IL-6 and TNF-α, coupled with upregulation of anti-inflammatory mediators like IL-10, estrogen helps maintain an optimal neuroinflammatory balance. This immune modulation is crucial for protecting neural circuits from inflammatory damage while supporting repair and adaptation mechanisms [29,30].

Progesterone’s influence on the nervous system represents a crucial adaptation mechanism during pregnancy, with implications extending beyond reproductive function, encompassing structural and functional modifications of neural circuits. During pregnancy, progesterone actively promotes myelin formation through the stimulation of oligodendrocyte progenitor cell differentiation. This enhanced myelination process involves increased expression of critical myelin proteins, particularly myelin basic protein, which supports efficient neural transmission during a period of heightened physiological demand [31].

The neurosteroid activity of progesterone manifests through its metabolites, such as allopregnanolone, which exerts powerful effects on neural function through GABA_A receptor modulation. During pregnancy, this mechanism helps maintain appropriate neuronal excitability thresholds. The anxiolytic and anticonvulsant properties of these neurosteroid metabolites serve as natural adaptations to protect maternal and fetal nervous systems from excessive neural activation, while modulating maternal stress responses [32].

The hormone establishes a balanced immune environment by inhibiting microglial activation and reducing pro-inflammatory mediator release. This immunomodulatory function becomes crucial in pregnancy, where maintaining appropriate inflammatory responses is essential for maternal neurological health and fetal neurodevelopment, supporting the significant physiological pregnancy-induced adaptations.

Progesteron’s neuroprotective mechanisms extend to central nervous system adaptations necessary for pregnancy and parturition. Progesterone influences neurotransmitter systems involved in pain perception, mood regulation, and autonomic control, all of which undergo significant modulation during pregnancy. Its effects on synaptic plasticity and neural circuit remodeling help prepare the maternal brain for the physiological and behavioral changes associated with pregnancy and motherhood [33].

Oxytocin is a sophisticated neuromodulator with widespread effects, vital for initiating and maintaining uterine contractions during labor. It is naturally released into the bloodstream during labor by the hypothalamus, stimulating the uterus to contract, and stored in the posterior pituitary gland. During pregnancy and the postpartum period, oxytocin enhances feelings of relaxation, trust, and emotional connection, reducing stress and promoting maternal behavior [34]. During pregnancy oxytocin’s modulation of the stress response system undergoes significant adaptation [35]. The hormone’s regulation of the hypothalamic–pituitary–adrenal axis becomes increasingly sophisticated as pregnancy progresses, creating a dampened stress response that protects both maternal and fetal development from excessive cortisol exposure. This modified stress response system helps maintain physiological and emotional homeostasis despite the significant challenges of pregnancy. The hormone helps modulate anxiety and mood fluctuations that commonly occur during gestation, while simultaneously enhancing emotional sensitivity to social cues that will be crucial for maternal care. This involves complex interactions with other pregnancy-related hormones, particularly progesterone and estrogen, which together modify neural circuits involved in emotional processing and social bonding. Oxytocin’s stress-buffering effects become particularly important as pregnancy approaches term. The hormone helps maintain emotional stability while simultaneously preparing the brain and body for the challenges of labor and delivery. This includes modifications to pain perception pathways and anxiety-processing circuits, creating a uniquely adapted neural state that supports the transition through labor to early motherhood.

Prolactin is a key regulator of neural plasticity, particularly during reproductive phases [36,37,38,39,40]. In the maternal brain, prolactin stimulates the formation of new neurons specifically in regions crucial for sensory processing and memory formation. The enhanced neurogenesis in the olfactory bulb improves the processing of olfactory signals crucial for offspring recognition, while hippocampal neurogenesis supports learning and memory adaptations necessary for maternal care. The mechanism of prolactin-induced neurogenesis involves complex cellular signaling cascades. Prolactin receptors, expressed in neural stem cells and progenitor populations, activate JAK/STAT pathways that regulate cell proliferation and differentiation. This signaling promotes the survival of newly generated neurons and their integration into existing neural circuits, particularly in regions involved in maternal behavior and memory formation. Its effects on protective instincts involve modulation of fear and anxiety circuits, particularly in the amygdala and related limbic structures. Prolactin helps calibrate the maternal brain’s threat assessment capabilities, enhancing vigilance toward potential dangers to offspring while maintaining appropriate stress responses that do not interfere with caregiving.

Cortisol modifies neural energy metabolism through multiple pathways, enhancing glucose availability to meet increased demands while simultaneously influencing neurotransmitter systems involved in cognitive function [41]. This metabolic modulation affects synaptic function and neural circuit efficiency, particularly in regions with high energy requirements such as the hippocampus and prefrontal cortex. Through membrane-bound receptors, cortisol can quickly modify neurotransmitter release and neural excitability, affecting immediate cognitive performance and emotional processing. The hormone’s genomic effects, mediated through nuclear receptors, lead to longer-term changes in gene expression that influence synaptic plasticity and neural circuit function. These alterations can significantly affect memory formation, emotional regulation, and executive function. Cortisol’s ability to cross the placental barrier represents a critical mechanism for normal development and potential pathology [42]. The hormone influences the development of key neural systems in the fetal brain, affecting the formation and refinement of circuits involved in stress response, emotional regulation, and cognitive function. The timing and magnitude of cortisol exposure become crucial factors, as they can permanently shape the developing neural architecture and influence future stress responsiveness. The hormone’s programming effects on fetal brain development involve epigenetic modifications that can persist throughout life. These changes affect the expression of genes involved in neural development, stress response system organization, and synaptic plasticity, potentially influencing long-term behavioral and cognitive outcomes.

**Figure 3:**
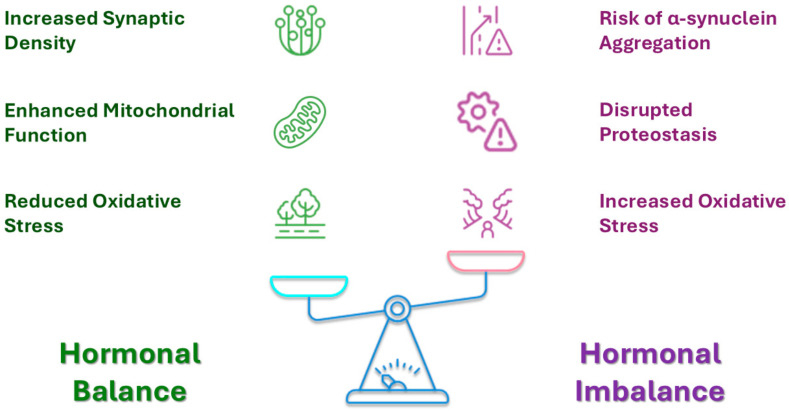
Balancing hormonal effects on neuronal health: illustration of hormonal influence on neurological conditions. Pregnancy-related hormones can exert protective effects on neuronal health, yet under conditions of imbalance, may increase vulnerability to neurodegenerative processes.

From the above, it is easy to conclude that pregnancy hormones like estrogen and progesterone can either protect brain cells or, under certain conditions, trigger or worsen protein misfolding involved in MSA. Figure 3 shows a simplified flowchart demonstrating how hormones impact neuronal health.

### 3.3. Immune System Modulation: Pregnancy-Induced Immunological Changes

Pregnancy is associated with a unique immunological state that balances tolerance to the semi-allogeneic fetus with the need to protect against pathogens. The immune system undergoes profound adaptations during pregnancy, characterized by sophisticated modulation of innate and adaptive immunity. Fetal microchimerism introduces another layer of complexity, with long-lasting effects on maternal immune regulation. In MSA, the interaction between fetal-derived cells and maternal neuroinflammation could exacerbate or modulate disease processes, particularly in pregnancies involving donor embryos achieved through ART.

A fundamental shift occurs in T helper cell responses, transitioning from Th1-dominated cell-mediated immunity toward Th2-predominant humoral immunity [43]. This shift manifests through enhanced production of Th2-associated cytokines, including IL-4, IL-5, and IL-10, while simultaneously reducing Th1 cytokines such as IFN-γ and IL-2. This altered immune profile has significant implications for autoimmune conditions, potentially ameliorating Th1-mediated pathologies while exacerbating Th2-associated allergic responses. Regulatory T cells (CD4+ CD25+ FoxP3+ Tregs) undergo significant expansion during pregnancy, establishing crucial immunological tolerance mechanisms [44,45]. These cells execute their regulatory functions through multiple pathways, including direct suppression of effector T-cell proliferation, modulation of antigen-presenting cell function, and secretion of immunosuppressive cytokines, particularly TGF-β [46]. This orchestrated regulation is essential for maintaining fetal tolerance while preserving maternal immune competence.

Natural killer cells in the decidua demonstrate distinct phenotypic and functional characteristics compared to their peripheral counterparts [47]. These decidual NK cells, characterized by CD56bright CD16- expression, contribute significantly to placental development and vascular remodeling. Their secretion of angiogenic factors, including VEGF, and specific cytokine profiles supports optimal fetal growth conditions while maintaining appropriate immune surveillance [48].

The dendritic cells modifications, including altered antigen presentation mechanisms that favor T-cell anergy or regulatory T cell differentiation, promote tolerogenic responses. The expression of specific surface molecules, notably HLA-G, undergoes modification to reduce immunogenicity while maintaining essential immune functions [49].

Hormonal regulation of immune responses during pregnancy involves complex interactions between estrogen, progesterone, and immune cell populations. These hormones influence immune cell development trajectories, cytokine production patterns, and adhesion molecule expression. Progesterone-induced blocking factor represents a crucial mediator of pregnancy-specific immune modulation, exhibiting effects on NK cell activity and cytokine production profiles.

The complement system undergoes careful regulation through the expression of specific regulatory proteins in decidual and trophoblast tissues. Proteins such as CD46, CD55, and CD59 provide essential protection against complement-mediated damage while maintaining appropriate immune surveillance capabilities [50].

**Figure 4:**
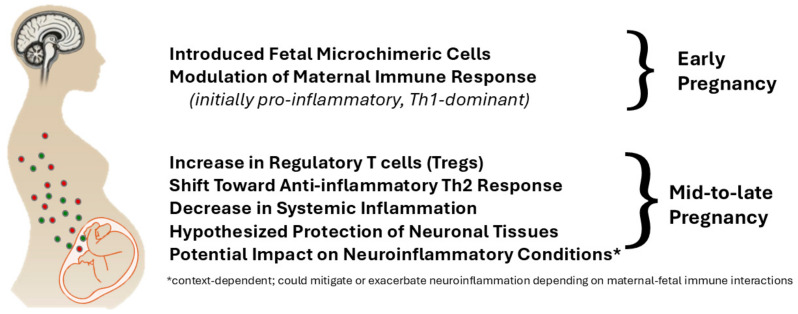
Microchimerism and immune modulation during pregnancy. The circles indicate hypothesized cytokine-mediated interactions between maternal and fetal immune systems, potentially affecting maternal neurological health, including MSA. Early pregnancy features a pro-inflammatory (Th1) immune environment with high NK cell activity. As pregnancy progresses, the immune response shifts toward an anti-inflammatory (Th2) profile, characterized by increased regulatory Treg activity, enhanced immune tolerance, and reduced systemic inflammation. Although generally protective, these immune adaptations in combination with fetal microchimerism could paradoxically influence maternal susceptibility to neuroinflammatory or neurodegenerative conditions. Following delivery, a postpartum immune recovery, marked by a reactivation of pro-inflammatory pathways, combined with the long-term persistence of fetal microchimeric cells in maternal tissues, may further contribute to immune dysregulation and neurological vulnerability.

Microchimerism, resulting from bidirectional cellular trafficking between mother and fetus, establishes long-term immunological consequences [51]. The impact extends beyond pregnancy due to the persistence of fetal cells in maternal tissues and maternal cells in offspring representing an intriguing aspect of pregnancy-induced immune modulation with implications for long-term health outcomes [52]. Fetal microchimerism, wherein fetal cells persist in maternal tissues, has been implicated in autoimmune disorders such as systemic lupus erythematosus and rheumatoid arthritis [53]. Similarly, studies suggest fetal microchimeric cells may traffic to maternal injury sites and differentiate into tissue-specific cells, such as cardiomyocytes, in response to damage [54]. These findings raise the possibility that fetal microchimerism might modulate neuroinflammatory and repair processes in neurodegenerative diseases, including MSA, particularly during pregnancy [55,56,57]. See Figure 4 for a simplified visual presentation of influence of fetal microchimerism on maternal immune modulation during pregnancy.

These immunological adaptations, while essential for successful pregnancy, have significant implications for concurrent pathological processes, particularly in the context of neurodegenerative conditions such as MSA. The modified immune responses may influence neuroinflammatory processes and the trajectory of neurodegeneration, necessitating careful consideration in clinical management strategies.

### 3.4. Pregnancy and Neurological Disorders

Pregnancy induces profound physiological, hormonal, and immunological adaptations that can modulate the course of various neurological conditions. While substantial research has focused on disorders like MS [58,59], which stems from autoimmune disorders where the immune system attacks myelin sheath of neurons, and, to a lesser extent, PD [60], a neurodegenerative disorder caused by the loss of neurons, the impact of pregnancy on other neurodegenerative conditions is less thoroughly studied. MS is an autoimmune disease, and pregnancy is known to modulate immune responses. While PD is not an autoimmune disease, there is growing evidence that neuroinflammation plays a role in its progression. Activated microglia and increased levels of inflammatory cytokines, e.g., TNF-α, IL-1β, are observed in PD, but these processes are secondary to neurodegeneration rather than the primary cause, as in MS. Nevertheless, examining MS and PD provides valuable context and comparative insights for understanding how pregnancy might influence a complex synucleinopathy such as MSA.

MS is a chronic, immune-mediated demyelinating disease of the central nervous system. One of the most consistently observed phenomena is that pregnancy, especially in the second and third trimesters, is associated with a reduced rate of MS relapses [61]. Estrogen, progesterone, and other pregnancy-associated hormones may exert immunomodulatory and neuroprotective effects, shifting the immune response toward a more tolerogenic state. This reduction in inflammatory activity frequently leads to clinical stabilization or improvement in MS symptoms during pregnancy. However, after delivery, when hormone levels revert to a pre-pregnancy baseline, there is a transient increased risk of relapse, particularly within the first three to six months postpartum. This pattern underscores the significant influence of hormonal and immunological shifts on disease activity. While pregnancy does not cure MS or halt its progression, it demonstrates that physiological changes can positively alter disease trajectory, at least temporarily [62].

Pregnancy does not appear to provide a pronounced neuroprotective or immunomodulatory environment for PD as it does for MS. However, PD, primarily characterized by dopaminergic neuronal loss in the substantia nigra and the presence of Lewy bodies (intraneuronal α-syn aggregates), is less common among women of childbearing age [63]. Consequently, data on the direct impact of pregnancy on PD are more limited. Small-scale observations and case reports suggest that pregnant women with PD may experience stable or variably fluctuating symptoms rather than consistent improvement or worsening. Hormonal changes might modestly influence dopaminergic function or neurochemical signaling, but no clear, reproducible pattern as in MS has emerged. Management complexities arise from the need to adjust PD medications-many of which are dopaminergic agonists or levodopa, due to teratogenic concerns. This can indirectly affect the stability of PD symptoms [60].

#### Comparative Analysis with MSA

Given the lack of documented cases of MSA pregnancies, this section extrapolates from known effects of pregnancy on neurological disorders, particularly PD [64], to hypothesize potential interactions between pregnancy-induced changes and MSA progression. MSA, like PD, is a synucleinopathy, but differs substantially in its underlying pathology and clinical presentation. MSA involves the accumulation of α-syn within oligodendrocytes, leading to widespread neurodegeneration affecting autonomic centers, the cerebellum, and the basal ganglia. Unlike MS, which is strongly influenced by immune-mediated mechanisms, MSA is considered a primarily sporadic disorder with more complex and less understood neuroinflammatory and glial dysfunction components [5]. While immune modulation plays a role in MSA pathology, it does not manifest as a relapsing-remitting disease with an overt inflammatory hallmark. Thus, the profound pregnancy-induced immunological tolerance that benefits MS patients may not confer equivalent advantage in MSA. While PD might present mild or stable symptom fluctuations during pregnancy, MSA involves widespread autonomic instability., e.g., severe orthostatic hypotension, urinary dysfunction and potentially rapid disease progression [63,65]. These features can be exacerbated by the hemodynamic and metabolic stresses of pregnancy. Since MSA generally occurs in older adults, instances of pregnancy affected by MSA are rarely documented, limiting the availability of empirical data. Nevertheless, the lack of a consistent immunologically mediated driver in MSA suggests that pregnancy’s immunomodulatory environment would not exert the stabilizing influence seen in MS [66]. Similarly, the complex neuropathology and higher severity level of autonomic compromise in MSA may overshadow any subtle hormonal neuroprotective effects that could, in theory, benefit other neurodegenerative conditions.

Table 3 highlights key insights in the comparison of MS and PD with MSA during pregnancy.

Pregnancy can have variable and condition-specific impacts on neurological disorders. While MS patients often experience reduced disease activity, PD’s symptom trajectory remains less predictable, and MSA’s rare intersection with pregnancy offers sparse data but fewer reasons to expect a beneficial modulation. This comparative lens reinforces the complexity of pregnancy’s influence on neurodegenerative conditions and underlines the importance of understanding each disorder’s pathophysiology. There is clearly a need for more research and case documentation in MSA to ascertain whether any elements of the pregnancy-induced physiological environment confer modest neuroprotective effects or simply become additional stressors on an already vulnerable nervous system.

## 4. Potential Links Between MSA and Pregnancy

### 4.1. Common Symptoms

**Figure 5:**
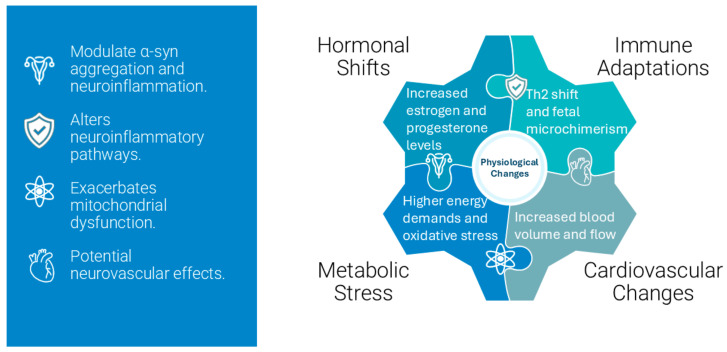
Key physiological changes during pregnancy, including hormonal fluctuations, immune adaptations, metabolic stress, and cardiovascular changes, interact with MSA pathophysiology. These changes may influence neuroinflammation, α-syn aggregation, and mitochondrial function, potentially altering disease progression.

During pregnancy, women undergo physiological and hormonal adjustments that can temporarily mimic or overlap with certain symptoms observed in MSA [69,70,71,72,73,74]. While the underlying mechanisms in MSA and pregnancy differ dramatically, the phenotypic convergence of these symptoms underscores the complexity of managing neurological conditions during pregnancy (Figure 5).

Orthostatic hypotension, an autonomic feature of MSA, is caused by the degeneration of autonomic pathways regulating vascular tone and blood pressure and characterized by a notable drop in blood pressure upon standing. During pregnancy, especially in the second and third trimesters, blood volume expands significantly and systemic vascular resistance may decrease, occasionally leading to transient dizziness or postural lightheadedness that, superficially, resembles the autonomic instability seen in MSA, with both conditions potentially showing similar patterns in blood pressure variability and baroreceptor sensitivity, complicating symptom assessment in pregnant women with existing or suspected neurodegenerative conditions [75,76].

Impaired bladder control in MSA often involves profound autonomic dysfunction resulting in urinary urgency, frequency, and incontinence [77]. Pregnancy, especially in the later trimesters as uterus expands, can exert mechanical pressure on the bladder and urethral sphincters, contributing to urinary frequency and, at times, mild incontinence—albeit typically reversible post-partum [78]. The distinction lies in the underlying mechanism: neurogenic bladder in MSA versus mechanical compression in pregnancy, though the clinical presentation may be remarkably similar. Likewise, chronic fatigue, a non-specific but burdensome symptom in MSA, can also afflict pregnant individuals, stemming from elevated metabolic demands, disturbed sleep patterns, and shifting hormonal profiles [79].

Sleep-related symptoms such as insomnia, disrupted sleep cycles, and non-restorative sleep are prevalent in MSA due to autonomic instability and neurodegenerative effects on sleep regulatory centers [80]. Pregnancy also predisposes individuals to disrupted sleep, whether due to physical discomfort, hormonal influences, or conditions like restless leg syndrome, which are more common during gestation [81]. Both conditions may affect rapid eye movement (REM) sleep behavior, though through different mechanisms: neurodegeneration of sleep centers in MSA versus hormonal and physical factors in pregnancy.

The pathophysiological causes differ markedly, with transient endocrine and mechanical factors in pregnancy versus neurodegenerative pathology in MSA; however, the shared phenotypic territory underlines the complexity of clinical interpretation [82]. This overlap becomes particularly challenging in cases where pregnancy might mask early MSA symptoms or when MSA-related symptoms might be mistakenly attributed to normal pregnancy changes. Such parallels highlight the importance of meticulous differential diagnosis and a nuanced understanding of how systemic physiological states seen in pregnancy may transiently approximate the symptomatic landscape of degenerative disorder. Recognizing that these symptoms, while common to both conditions, arise from distinct mechanisms is critical for appropriate management. This understanding can prevent misdiagnosis or overattribution of pregnancy-related physiological changes to an underlying neurodegenerative disorder, ensuring optimal care for mother and fetus.

#### Guidance for Differential Diagnosis

Due to symptomatic overlap, clinicians must carefully evaluate whether common pregnancy-related changes might be masking early signs of MSA. To assist in this differentiation, concise criteria are summarized in Table 4.

For clinical convenience, supplemental practical clinical recommendations for distinguishing MSA from typical pregnancy changes through targeted clinical assessments are summarized in Table 5.

These diagnostic recommendations emphasize early multidisciplinary collaboration to enhance maternal and fetal outcomes in suspected cases of MSA during pregnancy.

### 4.2. Age and Assisted Reproductive Technologies

The typical onset of MSA is between the fifth and sixth decade of life, placing its emergence well beyond the conventional childbearing window [83]. However, advances in ART have extended the maternal age range, enabling pregnancies to occur in women even in their early sixties [84,85,86]. As a result, a previously uncharted frontier emerges: pregnancies occurring within the biological timeframe when MSA typically manifests, potentially overlapping with the early disease course or prodromal phase of this neurodegenerative disorder. In addition, it alters clinical scenarios in which a patient may be carrying a pregnancy and exhibiting early neurological symptoms consistent with MSA. Changes in endocrine dynamics, autonomic function, and general homeostasis induced by pregnancy could interact with the underlying pathology of MSA, potentially exacerbating motor or autonomic symptoms or complicating differential diagnoses when new symptoms emerge during gestation.

The overlapping of ART and MSA presents unprecedented clinical scenarios that justifies careful consideration of multiple physiological systems. In donor embryo pregnancies achieved via ART in MSA patients, the maternal immune system faces a triple challenge: managing donor oocyte-derived antigens, donor sperm-derived antigens, and developing fetal antigens [87,88,89]. This triple antigenic exposure creates an immunological response with higher complexity than traditional pregnancies or single-donor in vitro fertilization (IVF) pregnancies. The complexity is further enhanced by the temporal dynamics of antigen presentation, as donor-derived components may persist long after the pregnancy, creating a sustained immunological challenge.

The presence of cells derived from two genetically distinct donors creates a unique form of complex microchimerism. Unlike traditional pregnancy-related microchimerism, this scenario involves three distinct genetic populations: maternal cells, donor-oocyte derived components, and donor-sperm derived components. This complex cellular mixture may circulate in maternal blood and potentially cross the blood–brain barrier (BBB), interacting with neural tissues affected by MSA. The integrity of the BBB itself may be compromised due to alterations in aquaporin-4 expression and function during pregnancy, potentially facilitating increased cellular trafficking to the central nervous system [90,91,92,93,94].

The immune system must maintain tolerance to multiple foreign antigens while managing the inflammatory processes associated with MSA. This creates a potentially heightened state of immune activation characterized by increased production of regulatory T cells to maintain tolerance to multiple foreign antigens, modified cytokine profiles responding to diverse antigenic stimuli, altered natural killer cell function dealing with multiple foreign cell populations, and enhanced dendritic cell activation processing various foreign antigens [95]. Amplified immune response may in turn influence neuroinflammatory impact and contribute to MSA pathology via increased BBB permeability due to enhanced inflammatory signaling, modified microglial activation patterns responding to diverse immune signals, altered cytokine profiles in the central nervous system, and enhanced oxidative stress due to sustained immune activation. In addition, the presence of multiple foreign antigens may trigger or modify autoimmune responses via molecular mimicry between foreign antigens and self-antigens, enhanced production of autoantibodies, modified T-cell responses to self-antigens, and altered immune regulatory mechanisms. For instance, increased levels of pro-inflammatory cytokines such as interleukin-6 (IL-6), tumor necrosis factor-alpha (TNF-α), and interleukin-1 beta (IL-1β), commonly associated with advanced maternal age and ART, may synergize with the neuroinflammatory pathways already implicated in MSA pathology. Additionally, elevated levels of IL-18 may directly influence α-syn aggregation, while specific chemokines such as CCL2 and CXCL10 may enhance inflammatory cell recruitment to the central nervous system [96,97]. The balance between Th1/Th2 cytokines becomes particularly crucial in donor embryo pregnancies, where the ratio may be altered by the presence of multiple foreign antigens [98,99].

Age-related changes in immune function further amplify the immunological ramification [100,101,102]. Advanced maternal age is associated with innate and adaptive immunity alterations, including reduced regulatory T-cell (Treg) function and increased pro-inflammatory cytokine production [103]. The diminished activity of Tregs compromises immune tolerance, potentially heightening the neuroinflammatory environment in MSA. Simultaneously, elevated levels of interferon-gamma (IFN-γ) and IL-17, key cytokines linked to age-related immune dysregulation, can exacerbate neuroinflammation through microglial priming and astrocytic activation, pathways critical in MSA pathology [104,105]. When combined with MSA-related neuroinflammation, this creates a potentially volatile immune environment that may influence disease progression and pregnancy outcomes.

Hormonal supplementation for ART and pregnancy maintenance becomes increasingly complex in women of advanced maternal age [106,107,108]. These interventions, necessary for successful implantation and early pregnancy support, may interact with MSA pathophysiology in previously undocumented ways. Human chorionic gonadotropin (hCG), beyond its role in pregnancy maintenance, may modulate immune responses and influence microglial activation patterns. Additionally, prolactin’s effects on microglial cells may either exacerbate or ameliorate neuroinflammation in MSA [109]. Exogenous estrogen, for example, has been shown to modulate α-syn expression and aggregation [110]. While some studies suggest estrogen may exert neuroprotective effects by dampening microglial activation and oxidative stress, others indicate that prolonged exposure to high estrogen levels can exacerbate protein misfolding and inflammatory signaling via nuclear factor kappa B (NF-κB) pathways [111,112,113,114]. The sustained administration of exogenous hormones, particularly estrogen and progesterone, could influence the immune response and neurodegenerative processes characteristic of MSA.

The MSA autonomic dysfunction becomes particularly challenging in the context of ART pregnancies. The cardiovascular adaptations required during pregnancy may be compromised by pre-existing autonomic insufficiency, while the hormonal and immunological changes associated with ART could potentially accelerate autonomic deterioration [115,116]. It creates a complex clinical scenario where standard pregnancy management protocols may require significant modification. For example, autonomic instability in MSA, often driven by impaired norepinephrine release and baroreflex dysfunction, may worsen during pregnancy due to expanded plasma volume and altered vascular resistance. The interconnection of autonomic failure with elevated circulating cytokines, such as IL-6, TNF-α, could further disrupt homeostatic cardiovascular responses, amplifying risks such as severe orthostatic hypotension or preeclampsia. Sympathetic denervation patterns characteristic of MSA may be particularly relevant to cardiovascular complications during pregnancy, while impaired thermoregulation may present additional challenges in managing pregnancy-related physiological changes [117].

The temporal overlap between MSA onset and ART-achieved pregnancy raises important questions about disease detection and progression. Early MSA symptoms might be masked by or confused with pregnancy-related changes, potentially delaying diagnosis. Conversely, the physiological stress of pregnancy might unmask or accelerate MSA progression in women with subclinical disease. This emerging clinical scenario necessitates careful monitoring of neurological and obstetric parameters throughout pregnancy, particularly concerning autonomic function and immune status. Notably, markers such as IL-6, IL-1β, and TNF-α may serve as potential indicators of pregnancy-related and neuroinflammatory processes, guiding clinical interventions. The management of these cases requires a multidisciplinary approach, integrating expertise in neurology, maternal-fetal medicine, and immunology to optimize outcomes for mother and fetus.

### 4.3. Physiological and Molecular Interactions

Pregnancy-induced physiological changes, including hormonal fluctuations and immune adaptations, have been shown to modulate disease trajectories in other neurological conditions such as PD. Applying these insights to MSA, this section explores hypothetical mechanisms that may underlie the interaction between pregnancy and MSA pathology. For instance, in multiple sclerosis, a shift toward a Th2-dominant immune profile leads to reduced relapse rates during pregnancy [118]. In PD, elevated estrogen levels have been suggested to enhance dopaminergic transmission, temporarily stabilizing symptoms. These examples suggest that pregnancy-induced hormonal changes could similarly impact MSA pathophysiology, though the direction and extent of their influence remain hypothetical [68].

Estrogen, a primary pregnancy hormone, operates through multiple neuroprotective pathways that could theoretically influence MSA progression. The hormone activates rapid non-genomic signaling via membrane-associated estrogen receptors, stimulating PI3K/Akt and MAPK cascades crucial for neuronal survival. These pathways enhance neuronal resilience through increased expression of antiapoptotic proteins while suppressing proapoptotic factors. Estrogen also influences synaptic plasticity by modulating dendritic spine morphology and density in brain regions critical for cognitive and autonomic function. This process involves upregulation of NMDA receptor subunit expression, particularly NR2B, and promotes the synthesis of essential synaptic proteins such as PSD-95 and synaptophysin. Furthermore, estrogen’s modulation of major neurotransmitter systems, including dopaminergic, serotonergic, and cholinergic pathways, directly influences neural circuits affected by MSA. However, the relationship between elevated pregnancy hormones and MSA progression presents a paradox. While estrogen’s antioxidant properties may potentially reduce oxidative stress in MSA-affected regions and maintain mitochondrial function, prolonged exposure to high hormone levels might exacerbate protein misfolding and enhance inflammatory signaling through NF-κB pathways. This dual nature of hormonal influence requires careful consideration in clinical management.

The immunological aspects of pregnancy introduce additional complexity to MSA pathophysiology through fetal microchimerism and immune system modulation. Fetal cells persisting in maternal tissues can differentiate into various cell types, including immune cells, potentially modifying the maternal immune environment and influencing neuroinflammatory processes associated with MSA. This biological phenomenon may have particular relevance in cases of donor embryo pregnancies achieved through ART, where complex microchimerism involving three distinct genetic populations could create unique immunological challenges. The maternal immune system undergoes substantial adaptation during pregnancy to maintain fetal tolerance while preserving defensive capabilities. This recalibration involves shifts in T-helper cell populations, altered cytokine profiles, and enhanced regulatory T-cell function. In MSA patients, these changes may interact with existing neuroinflammatory processes, potentially modifying disease progression through altered microglial activation patterns and cytokine production. The immune modulation becomes particularly relevant in the context of α-syn aggregation and neurodegeneration characteristic of MSA [53,54,55,56,57,119,120,121,122,123,124,125,126].

Pregnancy substantially increases metabolic demands and nutritional requirements, creating additional challenges for MSA patients. Enhanced nutrient demands, particularly for B-vitamins, iron, and essential fatty acids, may affect neurological function and potentially influence disease progression [127]. Proper nutrient allocation becomes crucial as the body balances fetal development needs with maternal neurological health. Oxidative stress emerges as a critical factor at this intersection. The increased metabolic rate during pregnancy generates higher levels of ROS, potentially exacerbating the oxidative damage already associated with MSA pathology [128,129,130,131]. This enhanced oxidative stress may accelerate α-syn aggregation and neurodegeneration through multiple mechanisms, including mitochondrial dysfunction and protein oxidation. Pregnancy-induced insulin resistance and altered glucose homeostasis may affect neural energy metabolism, particularly in regions already compromised by MSA pathology. These metabolic challenges may contribute to accelerated neurodegeneration through impaired cellular energy production and enhanced oxidative stress.

Understanding these molecular and physiological interactions provides crucial insights for clinical management strategies. The multifaceted influence of hormonal, immune, and metabolic factors necessitates careful monitoring and individualized treatment approaches. These considerations become particularly relevant in the context of advanced maternal age pregnancies achieved through ART, where the physiological challenges of pregnancy may interact with MSA pathology and age-related factors discussed in Section 4.2.

## 5. Case Study and Anecdotal Evidence

Due to its late onset, cases of MSA occurring during pregnancy are exceedingly rare, and the medical literature contains a single documented instance. Nevertheless, it will become increasingly important as ART-achieved pregnancies raise the bar of childbearing period, and improved diagnostic tools may detect MSA earlier. The reported case [65] represents the only documented instance of MSA during pregnancy, emphasizing the rarity of such scenarios and the need to extrapolate insights from Parkinson’s spectrum mechanisms to guide clinical hypotheses and research directions.

The case describes a successful full-term pregnancy in 35-year-old women with MSA, initially diagnosed at age 31 as dopa-responsive, idiopathic Parkinson’s disease, and confirmed MSA-P at age 39 pathologically. After caesarean section delivery of a female infant, the patient experienced transient improvement of symptoms post-delivery. The infant was healthy, with Apgar score nine at five minutes, weight 2.9 kg, and no neurological abnormalities. The child developed normally, except for dermoid cyst in the anterior neck and recurrent strabismus. The child was not breastfed.

Key takeaways from the case include potential exacerbation of autonomic symptoms, such as hypotension, urinary dysfunction, and gastrointestinal issues during pregnancy. The management of autonomic dysfunction during pregnancy presents unique challenges, particularly in maintaining cardiovascular stability and managing orthostatic hypotension. This highlights the connection between pregnancy-related physiological demands and pre-existing autonomic instability in MSA, underscoring the need to identify personalized care strategies based on the stage and severity of disease progression.

Anesthesia during labor poses a significant risk [132], particularly due to the unpredictable autonomic responses and potential complications with regional and general anesthesia, underscoring the necessity for a multidisciplinary care team to navigate challenges and devise a personalized care plan. This plan must account for the severity and progression of MSA symptoms while balancing the impacts on pregnancy and fetal development, including consideration of alternative therapies or adjusted dosing regimens. The transient symptom improvement observed post-delivery suggests the potential role of hormonal shifts, immune modulation, or reduced metabolic demands as transient modifiers of MSA pathology, a hypothesis demanding further investigation in controlled studies or future case series.

The challenge of medication management becomes particularly complex given the limited data on the safety of MSA treatments during pregnancy. Additionally, the overlap of MSA symptoms, such as fatigue, orthostatic hypotension, and urinary frequency, with normal physiological changes in pregnancy increases the risk of delayed diagnosis or misattribution of symptoms. This overlap underscores the importance of enhancing diagnostic criteria to distinguish MSA-specific autonomic and motor dysfunction from typical pregnancy-related symptoms. Tools such as advanced imaging, biomarker studies, or comprehensive autonomic testing could facilitate earlier and more accurate diagnoses.

Notably, the onset of symptoms in this patient at age 31 places her among the youngest reported cases of MSA. However, the frequency of post-mortem diagnosis in MSA raises the possibility that additional cases remain undocumented, particularly in women of childbearing age. Furthermore, it is conceivable that some cases of pregnancies recorded as PD might, in fact, represent undiagnosed MSA pregnancies, given the initial clinical overlap between early MSA and idiopathic PD. This diagnostic uncertainty is particularly relevant in younger patients, where the atypical age of onset might delay consideration of MSA as a differential diagnosis. Such misclassification highlights the importance of a systematic review of cases initially attributed to PD during pregnancy, with reevaluation using updated diagnostic criteria for MSA, to refine clinical understanding.

In this regard, it remains speculative whether pregnancy-associated mechanisms, such as hormonal modulation or metabolic stress, could contribute to accelerating or unmasking the underlying MSA pathology in women initially diagnosed with PD. Future research should explore how pregnancy-specific changes, such as estrogen-driven epigenetic modifications or altered α-syn aggregation dynamics, interact with disease mechanisms, potentially accelerating pathology in women at risk for MSA.

Historically, the temporal gap of MSA onset and conventional childbearing age meant that pregnancy and MSA rarely intersected, limiting clinical insights into their mutual impacts. However, with procedures such as IVF using donor egg cells redefining reproductive possibilities for women with diminished ovarian reserve or age-related infertility, the clinical possibilities are shifting. The case demonstrates the urgent need for standardized monitoring protocols for pregnant patients with MSA or early parkinsonism, integrating multidisciplinary expertise from neurology, obstetrics, and anesthesiology to anticipate complications. This evolving aspect necessitates enhanced surveillance and documentation of cases to build a knowledge base for future management protocols. Ongoing research and future case studies are essential to deepen the understanding of how MSA affects pregnancy, improve diagnostic accuracy in younger cohorts, and develop optimized management strategies for affected women.

## 6. Clinical Implications

**Figure 6:**
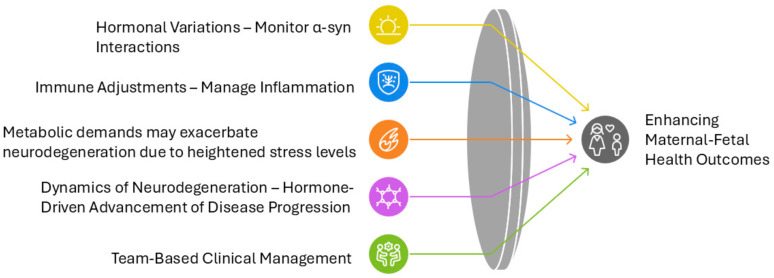
Understanding key clinical pathways for navigating MSA in pregnancy. The diagram illustrates five critical pathways converging to achieve optimal outcomes for mother and fetus.

The management of MSA during pregnancy presents unique clinical challenges that require carefully balanced approaches considering maternal disease progression and fetal safety (Figure 6). The rarity of MSA in reproductive-age women, combined with the increasing prevalence of advanced maternal age pregnancies through ART, necessitates specialized management protocols that address multiple aspects of care. Management strategies for pregnant MSA patients could draw on lessons from PD and MS, where pregnancy-related hormonal and immune adaptations have shown both protective and exacerbating effects on disease progression. Clinical insights from large-scale MS pregnancy registers [1,2,57,58,59,60,133] and PD literature [60,64,134] provide evidence-based analogies to support the speculative recommendations discussed here.

### 6.1. Diagnostic Challenges and Multidisciplinary Demands

The phenotypic overlap between pregnancy-related physiological changes and MSA symptoms creates significant diagnostic uncertainty. Common pregnancy symptoms such as orthostatic hypotension, urinary dysfunction, fatigue, and disrupted sleep patterns may mask or be confused with MSA manifestations, potentially delaying diagnosis. This challenge becomes particularly relevant in cases where pregnancy unmasks or accelerates previously subclinical neurodegenerative processes, especially in pregnancies achieved through ART where maternal age may align with typical MSA onset.

The complexity of MSA management during pregnancy demands integration of multiple medical specialties. A core team typically comprises a movement disorder neurologist, maternal-fetal medicine specialist, obstetric anesthesiologist, and physical medicine specialist. This team coordinates with rehabilitation specialists, nutritionists, and mental health professionals to provide comprehensive care addressing physical and psychological aspects of the condition.

### 6.2. Medication Management and Non-Pharmacological Interventions

Pharmacological management of MSA during pregnancy requires careful risk-benefit analysis for mother and fetus. Epigenetically targeted therapies, such as HDAC inhibitors, could theoretically offer neuroprotective benefits, but their safety during pregnancy remains to be established. Similarly, modulating miRNA profiles, particularly those associated with neuroinflammation, may represent a future therapeutic strategy for managing MSA in pregnant patients. Medications commonly used for autonomic dysfunction management, such as midodrine and fludrocortisone, carry Category C pregnancy ratings, necessitating careful consideration of their use [135,136]. Midodrine’s limited pregnancy data suggests restriction to cases where potential benefits clearly outweigh risks, while fludrocortisone use requires close monitoring for fluid retention and blood pressure changes. Droxidopa, another autonomic medication, lacks sufficient human pregnancy data, though animal studies indicate potential developmental effects [137,138,139].

For parkinsonian symptoms, levodopa emerges as a relatively safe option during pregnancy, though careful monitoring for orthostatic hypotension remains essential. Among dopamine agonists, bromocriptine offers a longer safety record in pregnancy. Monoamine oxidase B inhibitors generally warrant avoidance due to limited safety data [104,105,106,107,108,109,110,111,112,113,114,115,116,117,118,119,120,121,122,123,124,125,126,127,128,129,130,131,132,133,134,135,136,137,138,139,140,141]. Management of bladder dysfunction may employ oxybutynin, which holds a more favorable pregnancy safety profile compared to newer agents, but not in the first trimester [142]. When necessary, antidepressant treatment typically favors sertraline due to its well-documented safety profile during pregnancy [143].

Given the limitations of pharmacological approaches, non-pharmacological interventions assume critical importance in management strategies [144,145,146]. Nutritional interventions addressing oxidative stress and epigenetically influenced pathways, e.g., through methyl donors or antioxidant supplementation, could also play a role in mitigating disease progression. Physical therapy during pregnancy requires careful adaptation to address balance, mobility, and pelvic floor function while accounting for the changing maternal center of gravity and joint laxity. Supervised exercise programs must balance the benefits of maintained mobility against the risks of falls and autonomic decompensation. Lifestyle modifications play a crucial role in symptom management. The use of compression garments helps manage orthostatic hypotension, while elevation of the head during sleep can reduce nocturnal symptoms. Dietary adaptations, including small, frequent meals and careful hydration with electrolyte monitoring, help manage gastrointestinal symptoms while supporting maternal and fetal nutrition.

### 6.3. Immune System Modulation, Neuroinflammation, and Hormonal Influences on Neurodegeneration

Pregnancy induces profound immune tolerance mechanisms, including the expansion of Tregs and suppression of pro-inflammatory cytokines such as TNF-α, IL-1β, and IFN-γ [147,148,149]. While these adaptations promote fetal survival, their interaction with MSA-associated neuroinflammation remains unclear. Emerging evidence highlights the role of elevated IL-6 and other cytokines in recurrent miscarriage, preeclampsia and preterm delivery [150], and in exacerbating neurodegenerative processes via microglial priming and oxidative stress pathways. In women with MSA, the pregnancy-induced immune environment may either mitigate or amplify neuroinflammation, justifying close monitoring of inflammatory markers as potential indicators of disease progression. Fetal microchimerism during pregnancy introduces a potential source of persistent immune activation, which could intersect with epigenetically driven inflammatory pathways in MSA. Long-term monitoring of immune biomarkers may help guide therapeutic approaches.

Pregnancy-associated hormones exert neuroprotective and neuroimmune effects. However, prolonged or supraphysiological exposure to exogenous hormones, particularly during ART, may modulate α-syn aggregation and inflammatory pathways, for example via NF-κB activation. While estrogen’s neuroprotective effects may theoretically benefit MSA patients, its dual potential to exacerbate protein misfolding necessitates further investigation into safe hormonal supplementation protocols for women with neurodegenerative diseases [110,151,152,153,154,155,156].

### 6.4. Diabetes in Pregnancy and Potential Links to MSA

Pregnancy is associated with significant metabolic changes, including alterations in glucose homeostasis and increased insulin resistance. These adaptations, mediated in part by hormones such as prolactin, ensure an adequate supply of nutrients to the developing fetus but also increase the risk of gestational diabetes mellitus (GDM). GDM affects approximately 6–9% of pregnancies and is characterized by glucose intolerance that first appears or is first recognized during pregnancy [157,158,159,160,161,162].

Emerging evidence suggests a potential link between diabetes and neurodegenerative diseases, with some researchers proposing that conditions like Alzheimer’s disease and PD may represent a form of type 3 diabetes. The concept is based on the observation that insulin resistance and impaired glucose metabolism in the brain can contribute to the accumulation of misfolded proteins, oxidative stress, and neuroinflammation [163,164,165,166,167,168].

Hyperglycemia and insulin resistance associated with GDM may exacerbate the metabolic strain on the central nervous system, potentially contributing to the misfolding and aggregation of α-syn. Additionally, the pro-inflammatory environment induced by GDM could interact with the neuroinflammatory processes already implicated in MSA, further compromising neuronal function and survival. Prolactin has been shown to modulate microglial activation and neuroinflammation, suggesting a potential role in the progression of neurodegenerative diseases. However, the increased circulating levels of prolactin during pregnancy may influence glucose homeostasis and directly impact MSA pathology.

From a clinical perspective, these potential links underscore the importance of close monitoring and management of glucose levels in pregnant women with MSA. Regular screening for GDM, alongside careful control of blood sugar through diet, exercise, and medication when necessary, may help mitigate the potential exacerbation of MSA symptoms. Additionally, the postpartum period requires special attention, as the rapid hormonal changes and the resolution of insulin resistance may impact MSA progression.

### 6.5. Peripartum Considerations and Postpartum Management

The peripartum period requires exceptional attention to detail in care planning. Early anesthesia consultation allows development of individualized protocols accounting for autonomic dysfunction. Delivery planning must consider modified labor positions and emergency protocols for autonomic crises. The choice between regional and general anesthesia [132] requires careful evaluation of individual risk factors, with enhanced monitoring of blood pressure and autonomic function throughout the peripartum period.

Postpartum care necessitates vigilant monitoring of neurological and obstetric outcomes [20,34,59,133]. The immediate postpartum period requires careful fluid management and close observation of autonomic function. Gradual resumption of pre-pregnancy medications, if modified during gestation, must be balanced against the physiological changes of the postpartum period. Long-term management includes regular neurological assessments and ongoing physical therapy, with particular attention to supporting safe infant care activities within the context of progressive neurological disability.

### 6.6. Long-Term Maternal and Fetal Outcomes

While the documented case demonstrated a successful pregnancy outcome with a transient improvement in MSA symptoms postpartum, the long-term effects on maternal disease progression remain unknown. Additionally, the immunological and metabolic environment of pregnancy could, in theory, unmask subclinical MSA pathology or accelerate its course. From the fetal perspective, no direct evidence links maternal MSA to adverse neurological outcomes; however, the potential impacts of maternal autonomic dysfunction, hypoxia, or cytokine-mediated inflammation on fetal development require further investigation.

The convergence of advanced maternal age, ART, and neurodegenerative disease calls for longitudinal studies and case registries to better understand the clinical trajectory of MSA during pregnancy. Biomarkers such as circulating α-syn, IL-6, and TNF-α may provide insights into maternal neuroinflammation and fetal outcomes. Future research should focus on establishing evidence-based guidelines for managing autonomic dysfunction, hormone therapy, and anesthetic risks to improve maternal and fetal outcomes.

## 7. Discussion and Knowledge Gaps

### 7.1. Integrative Analysis

Pregnancy in MSA patients represents a complex biological scenario where molecular pathways, hormonal influences, and immune responses create a unique physiological environment. The integration of insights from molecular biology and gynecology reveals several key themes:

1. The potential for pregnancy-associated mechanisms to modify MSA progression through multiple pathways including hormonal modulation, immune system adaptation, and metabolic changes.
2. The role of ART in creating unprecedented biological scenarios, particularly in cases of donor embryo pregnancies where complex microchimerism may influence disease processes.
3. The challenge of distinguishing pregnancy-related physiological changes from MSA symptoms, particularly in early or prodromal disease stages.
4. The need for long-term studies to assess how pregnancy influences MSA progression and maternal outcomes, particularly in cases involving early-onset disease or ART-related pregnancies.
5. The prioritization of biomarker studies to identify pregnancy-specific indicators of disease activity, such as changes in α-syn levels, epigenetic modifications, or inflammatory markers.
6. The exploration of ART-related implications, such as how donor-derived microchimerism or ovarian stimulation protocols might intersect with neurodegenerative mechanisms in MSA patients.

These gaps underscore the need for interdisciplinary research to unravel the complex interactions between pregnancy and MSA, guiding both future clinical care and therapeutic development.

### 7.2. Research Gaps and Mechanisms

**Figure 7:**
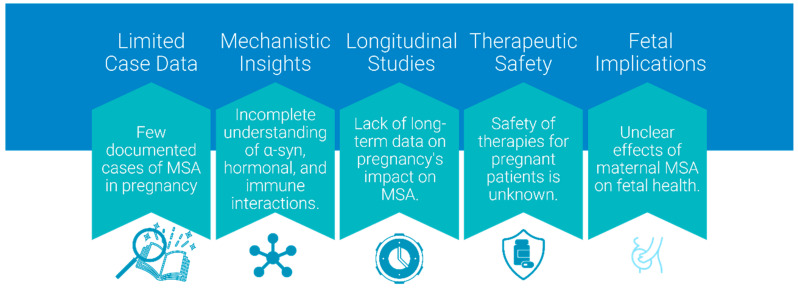
Critical research gaps in understanding the relationship between MSA and pregnancy include limited clinical data, mechanistic insights, and safety considerations for therapeutic approaches. Addressing these gaps is essential for advancing maternal and fetal care in this context.

Many mechanisms of MSA remain to be fully elucidated, and the interaction between MSA and pregnancy is even less understood due to the rarity of documented cases. While plausible, many proposed mechanisms, e.g., fetal microchimerism influencing MSA progression, remain speculative due to the scarcity of documented cases of pregnant MSA patients. However, analogous phenomena observed in related fields, such as autoimmune and neuroinflammatory conditions during pregnancy, provide a reasonable basis for these hypotheses and highlight the need for further research (Figure 7).

Pregnancy may complicate MSA mechanisms as hormonal changes, immune system modulation, lifestyle and environmental factors, and genetic and epigenetic influences may alter the course of pregnancy and impact the development of MSA. The scarcity of documented cases presents significant challenges in understanding the following: 1. the true prevalence of MSA in pregnancy, particularly in cases achieved through ART; 2. the long-term outcomes for mother and child; 3. the effectiveness of various management strategies; 4. the potential for pregnancy to modify disease progression.

Emerging evidence from related fields suggests that pregnancy may exert regenerative effects through unique biological mechanisms, such as hormonal modulation and fetal microchimerism. Studies have shown that pregnancy-associated hormonal shifts, particularly elevated estrogen and progesterone levels, can promote neuroplasticity, enhance synaptic connectivity, and stimulate oligodendrocyte progenitor cell differentiation, potentially improving neural repair processes. Additionally, fetal microchimerism, wherein fetal stem cells persist in maternal tissues and differentiate into various cell types, has been linked to tissue regeneration, such as heart muscle repair, and brain tissue repair in ischemic stroke of animal models [124,125,126]. While these effects remain underexplored in neurodegenerative diseases, it is plausible that similar mechanisms could offer a transient neuroprotective or regenerative influence in conditions like MSA. Such processes may counterbalance, even if temporarily, the oxidative stress and neuroinflammation characteristic of MSA. Investigating this potential avenue could open new perspectives on the intersection of pregnancy and neurodegenerative diseases, particularly through the lens of natural stem cell therapy effects mediated by pregnancy-specific factors.

Table 6 lists hypothetical mechanisms of mutual impact. The factors listed in Table 3 may encompass a broad range of hypothetical influences which may include potential triggers of MSA onset, exacerbation of pre-existing MSA or lead to complex interactions that accelerate underlying disease processes and unmask symptoms that were previously subclinical.

These hypothetical mechanisms underscore the complex relation between pregnancy and MSA pathophysiology, highlighting the need for further research to elucidate the specific pathways and interactions involved. By addressing these knowledge gaps, we can better understand the implications of pregnancy for MSA patients and develop evidence-based management strategies to optimize maternal and fetal outcomes.

### 7.3. Theoretical Models and Future Directions

Based on current understanding of MSA pathophysiology and pregnancy-related physiological changes, several theoretical models emerge regarding their interaction.

1. The “Double-Hit Hypothesis”: Pregnancy-related stress on cellular mechanisms might compound existing MSA pathology, potentially accelerating disease progression.
2. The “Protective Adaptation Model Extrapolated from Parkinson’s Spectrum Research”: Pregnancy-induced hormonal and immune adaptations, as observed in Parkinson’s spectrum research, could theoretically offer temporary protective effects against MSA progression, such as through enhanced neuroplasticity or reduced inflammation.
3. The “Advanced Maternal Age Risk Model”: The convergence of MSA-typical age of onset with ART-achieved pregnancies may create unique pathophysiological scenarios.

These theoretical frameworks provide direction for future research efforts and clinical observation. Priority areas for investigation should include 1. longitudinal studies of MSA patients achieving pregnancy through ART; 2. molecular studies of α-syn behavior under pregnancy-associated conditions; 3. development of specialized monitoring protocols for pregnant MSA patients; 4. investigation of optimal management strategies balancing maternal and fetal needs.

### 7.4. Maternal-Fetal Transmission Considerations

MSA exhibits prion-like characteristics. The misfolding process of α-syn proteins shares similarities with prion diseases, where abnormal proteins propagate by inducing misfolding in normal counterparts. Research has shown that misfolded α-syn can spread from cell to cell within the brain, contributing to the progression of neurodegeneration. However, this propagation is mostly limited to the individual’s nervous system and does not imply transmissibility between individuals. MSA is considered a sporadic neurodegenerative disorder with no known cases of vertical transmission, i.e., from parent to offspring, documented in the medical literature. In pregnant patients with MSA, the placenta acts as a selective barrier between the maternal and fetal circulatory systems, with no evidence that misfolded α-syn proteins involved in MSA can cross it. Although some proteins and antibodies can cross this barrier, even in classical, more infectious prion diseases like Creutzfeldt–Jakob Disease (CJD), there is no documented evidence of mother-to-child transmission during pregnancy or childbirth.

However, the question at hand is whether misfolded α -syn proteins, potentially carried in extracellular vesicles (EVs) released by cells, including neurons and glial cells, could cross the placental barrier and affect the fetus. EVs, such as exosomes can contain misfolded α-syn, and facilitate the spread of pathology by transferring misfolded proteins to healthy cells, inducing aggregation . Exosomes containing misfolded α-syn, may cross the BBB and enter systemic circulation. Theoretically, EVs present in maternal circulation could reach the fetus. However, animal studies often show that neurodegenerative disease proteins do not cross the placental barrier in significant amounts [181]. That could be due to multiple protective mechanisms that go beyond placental barrier effciency, including immune defenses which neutralize or degrade foreign proteins and vesicles, and fetal cellular protections which demonstrate intrinsic mechanisms to maintain protein homeostasis and prevent the aggregation of misfolded proteins. While theoretical models can propose potential risks, empirical evidence to date supports the conclusion that protective mechanisms effectively prevent the transmission of MSA from mother to child (Table 7).

Several protective mechanisms exist to prevent the transfer of misfolded α-syn proteins across the placental barrier. The placenta acts as a highly selective filter, with tight junctions between trophoblast cells and active transport systems that regulate the passage of substances. The size and physicochemical properties of misfolded α-syn aggregates may preclude their efficient crossing of this barrier. Furthermore, the placenta possesses a unique immunological environment characterized by the presence of specialized macrophages (Hofbauer cells) and natural killer cells that can recognize and eliminate foreign or abnormal proteins [183]. The placental expression of enzymes like proteases and peptidases may also contribute to the degradation of misfolded proteins.

In addition to placental defenses, the developing fetus possesses its own cellular mechanisms to maintain protein homeostasis and prevent the aggregation of misfolded proteins. These include robust heat shock protein expression, efficient proteasomal degradation, and active autophagy pathways. The fetal BBB and the unique developmental environment of the fetal brain may also limit the potential for misfolded α-syn to propagate pathology.

While the theoretical possibility of maternal-fetal transmission of misfolded proteins cannot be entirely excluded, the multiple layers of protection at the placental interface and within the fetal environment itself likely minimize this risk. Continued research into the specific mechanisms of protein transfer and the developmental resilience of the fetal brain will be crucial for further elucidating the potential for maternal-fetal transmission in the context of neurodegenerative disorders like MSA.

MSA is predominantly sporadic with a very limited genetic component. Unlike some prion diseases that have familial forms due to specific genetic mutations, no definitive hereditary patterns have been established for MSA. Also, no evidence is present indicating that MSA mechanisms can remain dormant in an individual from birth and become active later in life due to maternal transmission during pregnancy. The expression levels of α-syn in the fetal brain are different from those in adults. Even if misfolded α-syn were somehow present, the fetal environment does not favor the pathological aggregation processes seen in MSA. The fetal immune system and cellular quality control mechanisms, such as proteasomal degradation and autophagy, are adept at handling misfolded proteins, reducing the likelihood of protein aggregation and neurodegeneration.

### 7.5. Limitations of Current Research and Ethical Considerations

While this review synthesizes current knowledge and explores hypothetical scenarios based on comparative diseases such as PD and MS, significant limitations remain due to the rarity and lack of direct documentation of MSA during pregnancy. The speculative nature of current hypotheses highlights the critical need for empirical data, validated diagnostic criteria specific to MSA during pregnancy, systematic clinical studies, and dedicated case registries. Recent advances in diagnostic biomarkers are continually improving our ability to detect MSA at earlier stages, potentially identifying dormant phases of the disease. Indeed, the documented post-mortem diagnosis of MSA in a patient as young as 31 suggests that improved diagnostic accuracy may reveal additional early-onset cases, previously unrecognized due to diagnostic limitations. Future research utilizing these enhanced diagnostic tools could offer critical insights into triggers initiating or exacerbating MSA progression. Such advances would clarify the disease’s pathophysiological onset and raise awareness about the necessity for broader, collaborative clinical research efforts, ultimately guiding evidence-based guidelines and optimizing maternal-fetal outcomes.

Beyond clinical and research challenges, women diagnosed with or at risk of MSA who consider pregnancy, especially through ART, may face substantial ethical and social dilemmas. Potential issues include difficult decisions regarding pregnancy continuation or termination, influenced by uncertainties around disease prognosis, the effectiveness and sensitivity of diagnostic tests, and concerns about maternal and fetal well-being. Additionally, limited awareness among healthcare professionals about MSA could delay diagnosis or result in inadequate counseling, complicating informed decision making for affected individuals. These challenges underscore the need for enhanced multidisciplinary support systems that incorporate medical, ethical, psychological, and social perspectives to comprehensively address the unique needs of women navigating pregnancy in the context of MSA.

## 8. Emerging Challenges and Future Directions in the Era of ART

Pregnancy in MSA patients represents an emerging clinical challenge that demands careful consideration as ART extend childbearing possibilities into later decades of life. This review has elucidated critical molecular and clinical interactions while identifying significant knowledge gaps that warrant further investigation.

The physiological changes of pregnancy can significantly impact MSA progression through multiple mechanisms. Hormonal fluctuations, particularly involving estrogen and progesterone, may influence α-syn aggregation and neurodegeneration via protective and potentially harmful pathways. The immune modulation characteristic of pregnancy, including the phenomenon of fetal microchimerism, creates a unique biological environment that may interact with MSA’s neuroinflammatory components. While fetal microchimerism and hormonal influences are proposed as potential modulators of MSA progression during pregnancy, there is limited direct evidence. Insights from other neurodegenerative and autoimmune conditions suggest these mechanisms could be relevant, but targeted studies are needed to validate their role in MSA. This highlights a broader gap in understanding how pregnancy-induced physiological changes interact with neurodegenerative diseases.

The metabolic and autonomic demands of pregnancy can unmask or exacerbate underlying MSA pathology, especially in cases of previously undiagnosed or early-stage disease. For healthcare providers managing pregnant patients with MSA, several key recommendations emerge from current evidence. First, early recognition of MSA symptoms in pregnant patients of advanced maternal age requires heightened vigilance, particularly given the overlap between pregnancy-related changes and MSA manifestations. Second, management strategies should employ a multidisciplinary approach, carefully balancing medication choices with pregnancy safety considerations while maximizing non-pharmacological interventions. Third, particular attention must be given to peripartum planning, including careful consideration of anesthetic approaches and delivery methods that account for autonomic dysfunction.

The phenomenon of complex microchimerism in donor embryo pregnancies, involving three distinct genetic populations, may create immunological scenarios requiring careful monitoring and specialized management approaches. Healthcare providers must remain vigilant for potential interactions between the donor-derived cellular components and MSA pathophysiology.

Critical gaps in current knowledge highlight several priorities for future research. Longitudinal studies tracking MSA progression during and after pregnancy are essential to understand the long-term implications of this intersection. Molecular studies investigating the impact of pregnancy-related hormonal and immune changes on α-syn aggregation and neuroinflammation could provide valuable insights into disease mechanisms. Development of specialized protocols for monitoring and managing pregnant MSA patients, particularly those achieving pregnancy through ART, represents an urgent clinical need. The convergence of MSA and pregnancy, while currently rare, may become increasingly common as reproductive technologies advance. This emerging clinical scenario demands continued attention from researchers and clinicians to optimize outcomes for this unique patient population. Future investigations should focus on elucidating the molecular mechanisms underlying this interaction while developing evidence-based guidelines for clinical management. As our understanding of MSA pathophysiology and the biological complexities of advanced maternal age pregnancy continues to evolve, the importance of documenting and studying cases of MSA in pregnancy becomes increasingly apparent. This knowledge will be crucial for developing targeted interventions and management strategies that address the specific challenges faced by this patient population.

**Figure 8:**
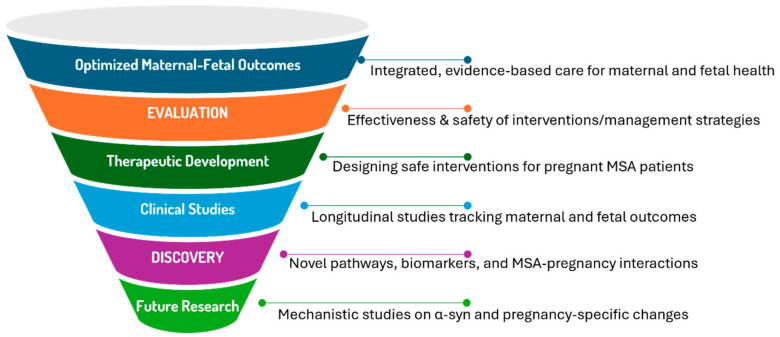
A proposed framework for addressing the interplay of MSA and pregnancy, emphasizing a stepwise approach from foundational research to optimized maternal-fetal outcomes. The framework includes discovery of novel pathways and biomarkers, clinical studies, therapeutic development, and evaluation to inform evidence-based care.

This review has highlighted the potential molecular and physiological interactions between MSA pathophysiology and pregnancy-related changes, emphasizing the need for heightened clinical alertness and a multidisciplinary approach to management. The unprecedented biological scenarios introduced by donor embryo pregnancies and the phenomenon of complex microchimerism underscore the importance of continued research and clinical documentation. Key priorities for future investigation include longitudinal studies tracking MSA progression during and after pregnancy, molecular explorations of the impact of pregnancy-related hormonal and immune changes on α-syn aggregation and neuroinflammation, and the development of specialized protocols for monitoring and managing pregnant MSA patients (Figure 8).

As the frontiers of reproductive medicine continue to expand, the intersection of neurodegenerative disorders like MSA and pregnancy is likely to become increasingly relevant. By extrapolating insights from Parkinson’s spectrum research, this review highlighted the potential for pregnancy-induced hormonal and immune adaptations to influence MSA progression, emphasizing the urgent need for targeted studies and clinical collaboration to explore and optimize outcomes for this unique patient population.

## Figures and Tables

**Table 1:** Glossary of key terms.

Term	Simplified Definition
Multiple System Atrophy (MSA)	A rare neurological disorder affecting movement, balance, and autonomic functions due to nerve cell damage.
α-Synuclein (α-syn)	A protein commonly found in brain cells that, when misfolded, forms harmful aggregates causing nerve damage.
Glial Cytoplasmic Inclusions (GCIs)	Abnormal clusters of misfolded α-syn within support cells (oligodendrocytes) in the brain, a hallmark of MSA.
Synucleinopathies	A group of neurodegenerative disorders characterized by the buildup of α-syn protein, including MSA and Parkinson’s disease.
Microchimerism	Presence of a small number of cells originating from another individual, genetically distinct from the host.
Fetal Microchimerism	Presence and persistence of fetal cells within maternal tissues, which can influence maternal immune responses.
Epigenetics	Changes in gene expression without altering the DNA sequence, including mechanisms like DNA methylation, histone modifications, and non-coding RNAs.
Neuroinflammation	Inflammation in the nervous system that may contribute to nerve damage or degeneration.
Reactive Oxygen Species (ROS)	Chemically reactive molecules containing oxygen that can damage cells if produced excessively.
Oxidative Stress	A harmful state resulting from excessive ROS, damaging cells and contributing to disease progression.
Exosomes	Small vesicles released by cells, carrying proteins, RNAs, and other molecules.
	facilitating cell communication by transferring molecules, including misfolded proteins.
Autophagy	A cellular process of recycling damaged or unnecessary components to maintain cellular health.
Proteostasis	Regulation of cellular protein balance to ensure proper folding and function.
T helper (Th) cells (Th1, Th2)	Immune cells helping regulate immune responses. Th1 promotes inflammation. Th2 supports antibody production and reduces inflammation.
Regulatory T cells (Tregs)	Specialized immune cells that suppress excessive immune responses and maintain tolerance to self and fetal tissues.
Natural Killer (NK) Cells	Immune cells crucial for early defense, particularly in pregnancy, aiding placental development and maternal-fetal immune tolerance.
Dendritic Cells	Immune cells that capture and present antigens, coordinating immune responses.
Hormonal Modulation	Adjustments of body functions and immune responses by hormones such as estrogen, progesterone, hCG, prolactin, or oxytocin.
Assisted Reproductive Technologies (ART)	Medical techniques like in vitro fertilization (IVF) used to assist in achieving pregnancy.

**Table 2:** Mechanism and factors relevant to MSA.

Neurodegeneration Mechanisms [3,5,7,10]	Genetic Predispositions [5,9]	Epigenetic Modifications [5,8]
Mitochondrial Dysfunction	SNCA Gene Variants	DNA Methylation
Oxidative Stress	COQ2 Mutations	Histone Modifications
Neuroinflammation	MAPT and Other Genes	Non-Coding RNAs
Proteostasis Impairment	Copy Number Variations	Environmental Epigenetics
Iron Homeostasis Dysregulation		
Cell-to-Cell Transmission of Pathology		

**Table 3:** Comparative overview of pregnancy-related effects on MS, PD, and MSA.

Aspect	MS	PD and MSA
Hormonal and Immune Interactions	Gains the most from pregnancy-induced immunotolerance, reducing relapse rates and demonstrating temporary modulation of disease activity [66].	May not benefit significantly from hormonal shifts. PD shows no consistent pregnancy-related remission, and MSA’s complex pathology and older onset age limit such effects [60,65].
Disease Mechanism Alignment	Strong inflammatory component aligns well with the immunological adaptations of pregnancy [66].	Primarily neurodegenerative with misfolded protein accumulation. Potential pregnancy-related benefits would need to come from metabolic, vascular, or subtle neuroprotective changes rather than immunological tolerance [60,63].
Treatment and Medication Considerations	Pregnancy can reduce the need for high-intensity immunotherapies [67].	Medication regimens may need careful adjustment to ensure maternal-fetal safety; the limited range of safe treatments can complicate management [65,68].

**Table 4:** Criteria for differentiating MSA symptoms from normal pregnancy-associated symptoms.

Symptom	Normal Pregnancy	Potential MSA Indicator
Orthostatic Hypotension	Mild dizziness, brief and self-resolving upon standing, common in later trimesters due to increased vascular volume and reduced vascular resistance.	Severe and persistent dizziness upon standing; significant blood pressure drops (>30 mmHg systolic); symptoms not improved by typical hydration and positional adjustments.
Urinary Frequency/Incontinence	Typically mild, progressively worse during pregnancy, predominantly mechanical due to pressure from the uterus, improving after delivery.	Significant, progressive urinary urgency and frequency; potential incomplete bladder emptying or retention due to autonomic dysfunction rather than mechanical pressure; symptoms persist or worsen postpartum.
Fatigue	Usually improves with rest; primarily related to increased metabolic demands and sleep disruption.	Severe and unrelenting fatigue disproportionate to pregnancy stage, minimally responsive to rest, possibly associated with increasing autonomic dysfunction, e.g., fluctuating blood pressure, sleep apnea.
Sleep Disturbances	Mild disturbances mainly from physical discomfort, hormonal changes, and transient conditions, such as restless leg syndrome.	Persistent or progressive severe disturbances, possibly including REM sleep behavior disorder, autonomic dysfunction such as sudden fluctuations in blood pressure/heart rate during sleep, or sleep apnea.
Motor Symptoms	Minimal, such as occasional mild clumsiness or balance issues associated with altered center of gravity during pregnancy.	Noticeable progressive deterioration in motor coordination, stiffness, slow movements (parkinsonism), tremors, or gait instability unrelated to pregnancy changes.

**Table 5:** Recommended clinical actions for differential diagnosis in suspected MSA during pregnancy.

Clinical Action	Recommended Use Case/Indications
Monitoring and Documentation	Maintain detailed symptom diaries documenting symptom progression, severity, and persistence relative to pregnancy stages.
Autonomic Testing	Conduct active stand or tilt-table testing if orthostatic hypotension symptoms exceed typical pregnancy-associated dizziness or if severe blood pressure drops occur.
Urodynamic Assessment	Perform urological evaluation if urinary symptoms such as frequency, urgency, and retention persist or worsen, particularly postpartum.
Sleep Studies	Evaluate patients with persistent severe sleep disturbances, such as suspected REM sleep behavior disorder or sleep apnea, beyond normal pregnancy discomfort.
Neurological Consultation	Initiate multidisciplinary consultation, e.g., neurology, and maternal-fetal medicine, early if symptoms appear progressive, severe, atypical, or resistant to typical management strategies.

**Table 6:** Hypothetical mechanisms impacting MSA in pregnant patients.

Mechanism	Concept	Hypothetical Link
Fetal Microchimerism and Immune Modulation [169]	Fetal cells migrate into the maternal body and persist for years.	Could influence maternal immune responses. If MSA has an autoimmune aspect, fetal microchimerism might exacerbate MSA symptoms by altering immune regulation.
Pregnancy-Induced Neuroplasticity [163,170]	Pregnancy induces significant neuroplastic changes in the brain.	Neurological adaptations might, in rare cases, interact adversely with MSA pathology, accelerating neurodegeneration due to altered neural circuitry.
Altered Cerebral Blood Flow [164,165]	Pregnancy increases blood volume and alters cerebral flow.	Vascular changes could unmask or worsen MSA symptoms by affecting blood supply to key brain regions involved in autonomic and motor functions.
Endogenous Retroviruses Activation [166]	Pregnancy can activate endogenous retroviruses (ERVs).	ERV activation may theoretically promote inflammation or protein misfolding, potentially contributing to MSA-related neurodegenerative processes.
Nutrient Depletion and Neurodegeneration [167,168,171,172]	Pregnancy heightens nutritional demands.	Deficiencies in critical nutrients (iron, B-vitamins, omega-3s) could, speculatively, exacerbate neurodegeneration or trigger MSA onset.
Metabolic Stress and Protein Aggregation [129,173,174,175]	Pregnancy increases metabolic and oxidative stress.	Heightened oxidative stress might encourage α-synuclein aggregation, linking metabolic demands of pregnancy to MSA pathology.
Placental Transfer of Environmental Toxins [174]	Placenta can transfer certain toxins to the fetus.	Neurotoxin accumulation in maternal tissues might influence MSA development in the mother or increase the child’s future risk of neurodegenerative conditions.
Hormonal Influence on α-Syn Expression [176]	Hormonal fluctuations occur during pregnancy.	Shifts in estrogen and progesterone could affect α-syn production or aggregation, potentially impacting MSA pathology.
Gut-Brain Axis Alterations [177,178]	Pregnancy alters gut microbiota composition.	Changes in the microbiome could affect neural signaling or immune responses, perhaps contributing to MSA progression.
Psychological Stress and Epigenetic Changes [179,180]	Pregnancy-related stress leads to epigenetic modifications.	Stress-induced epigenetic changes might activate neurodegenerative genes or suppress protective pathways, linking pregnancy stress to MSA onset or exacerbation.
Donor Embryo Pregnancies via ART [119]	Triple immune challenge	Complex microchimerism with three distinct genetic populations may interact with neural tissues affected by MSA, creating a potentially heightened state of immune activation, autoimmune modulation, and neuroinflammatory impact.
Pregnancy-Associated Hormonal Influence on Epigenetic Pathways [120,121,122,123]	Hormonal modulation of histone deacetylation or miRNA expression could potentially mitigate α-syn expression and aggregation.	Pregnancy-associated hormonal changes, such as elevated estrogen levels, are known to influence DNA methylation and histone modification. These changes could modulate the expression of α-syn and neuroinflammatory mediators, creating a dynamic interaction between pregnancy and MSA pathophysiology.
Natural stem cell therapy effect of microchimerism [54,124,125,126]	Fetal microchimerism has been linked to maternal tissue regeneration.	Fetal cells persist in maternal tissues and differentiate into various cell types.

**Table 7:** Protective mechanisms preventing fetal transmission of misfolded α-Syn.

Mechanism	Description
Placental Barrier’s Selectivity	Structural Barriers: The placenta consists of multiple layers (syncytiotrophoblasts, cytotrophoblasts) that physically prevent the passage of large molecules and vesicles. Active Transport Mechanisms: Transport across the placenta often requires specific receptor-mediated pathways; exosomes may lack ligands needed for uptake [182].
Immune Surveillance in the Placenta	Placental Immune Cells: Hofbauer cells (macrophages) within the placenta can phagocytose and degrade foreign particles, including exosomes. Toll-Like Receptors (TLRs): Trophoblasts express TLRs that recognize PAMPs and trigger innate immune responses against harmful exosomal content [183].
Enzymatic Degradation	Proteases in the Placenta: Placental enzymes degrade proteins and nucleic acids within exosomes, reducing the likelihood of intact α-syn transmission [184].
Limited Expression of Receptors	Exosomal Uptake Receptors: Fetal cells may express fewer receptors necessary for internalizing maternal exosomes, limiting their uptake [114].
Fetal Immune System	Developing Immunity: Despite immaturity, the fetal immune system can recognize and degrade foreign proteins. Autophagy pathways help clear misfolded proteins that cross the placental barrier [185,186,187].
Lack of Prion Protein Interactions	Prion-Like Mechanisms: Misfolded α-syn propagates via templating normal α-syn. Fetal neurons may express lower levels of α-syn or lack critical co-factors, preventing prion-like propagation [188,189,190].
Efficient Placental Filtering	Size Exclusion: Exosomes’ size may restrict their ability to cross the placental barrier. Selective Permeability: The placenta allows passage of essential nutrients and gases while excluding larger complexes [191].
Maternal Immunoglobulin Regulation	IgG Transfer: Maternal IgG antibodies cross the placenta to provide passive immunity; there is no evidence that α-syn or other pathogenic proteins can utilize this pathway [192].
Autophagy and Proteostasis in Fetal Cells	Robust Proteostasis Mechanisms: Fetal cells possess active autophagy and proteasomal pathways to degrade misfolded proteins. Chaperone Proteins: Molecular chaperones in fetal cells prevent the aggregation of misfolded proteins [193].
Epigenetic Safeguards	Gene Expression Regulation: Epigenetic mechanisms can downregulate genes that promote α-syn aggregation. Protective MicroRNAs: Specific fetal microRNAs inhibit α-syn translation or promote its degradation [194].
Bioelectric Repair	Inherent plasticity of developing tissues maintains intended morphogenetic blueprint. Robust bioelectric gradients at the placental barrier and within fetal tissues ensure structural integrity, selective permeability, and resilience against prion-like propagation [186,195].

## Data Availability

No new data were created or analyzed in this study.
